# Na^+^ current expression in human atrial myofibroblasts: identity and functional roles

**DOI:** 10.3389/fphys.2014.00275

**Published:** 2014-08-07

**Authors:** Jussi T. Koivumäki, Robert B. Clark, Darrell Belke, Colleen Kondo, Paul W. M. Fedak, Mary M. C. Maleckar, Wayne R. Giles

**Affiliations:** ^1^Simula Research Laboratory, Center for Biomedical Computing and Center for Cardiological InnovationOslo, Norway; ^2^Faculty of Kinesiology, University of CalgaryCalgary, AB, Canada; ^3^Division of Cardiothoracic Surgery, Department of Cardiac Sciences, University of CalgaryCalgary, AB, Canada

**Keywords:** Na^+^ current, atrial arrhythmias, mathematical models, fibroblast, myofibroblast

## Abstract

In the mammalian heart fibroblasts have important functional roles in both healthy conditions and diseased states. During pathophysiological challenges, a closely related myofibroblast cell population emerges, and can have distinct, significant roles. Recently, it has been reported that human atrial myofibroblasts can express a Na^+^ current, I_Na_. Some of the biophysical properties and molecular features suggest that this I_Na_ is due to expression of Na_v_ 1.5, the same Na^+^ channel α subunit that generates the predominant I_Na_ in myocytes from adult mammalian heart. In principle, expression of Na_v_ 1.5 could give rise to regenerative action potentials in the fibroblasts/myofibroblasts. This would suggest an active as opposed to passive role for fibroblasts/myofibroblasts in both the “trigger” and the “substrate” components of cardiac rhythm disturbances. Our goals in this preliminary study were: (i) to confirm and extend the electrophysiological characterization of I_Na_ in a human atrial fibroblast/myofibroblast cell population maintained in conventional 2-D tissue culture; (ii) to identify key molecular properties of the α and β subunits of these Na^+^ channel(s); (iii) to define the biophysical and pharmacological properties of this I_Na_; (iv) to integrate the available multi-disciplinary data, and attempt to illustrate its functional consequences, using a mathematical model in which the human atrial myocyte is coupled via connexins to fixed numbers of fibroblasts/myofibroblasts in a syncytial arrangement. Our experimental findings confirm that a significant fraction (approximately 40–50%) of these human atrial myofibroblasts can express I_Na_. However, our data suggest that I_Na_ may be generated by a combination of Na_v_ 1.9, Na_v_ 1.2, and Na_v_ 1.5. Our results, when complemented with mathematical modeling, provide a background for re-evaluating pharmacological management of supraventricular rhythm disorders, e.g., persistent atrial fibrillation.

## Introduction

Within approximately the last 10 years, experimental results and clinical data have established that both fibroblast and myofibroblast cell populations in the mammalian heart (including the adult human) can play very important functional roles in physiological and pathophysiological settings, respectively (Nattel, 2002; Baudino et al., 2006; Munoz et al., 2008; Porter and Turner, 2009; Baum and Duffy, 2011; Biernacka and Frangogiannis, 2011). The fibroblast population, when judged in terms of cell numbers, is predominant in all adult mammalian hearts (cf. Baudino et al., 2006). Until quite recently, these fibroblasts were considered to be passive. That is, they were thought to have few, if any primary electrophysiological roles. Rather, both fibroblasts and myofibroblasts were believed to function predominantly in the synthesis, and degradation (homeostasis) of the extracellular matrix, largely as a consequence of their roles in collagen production/breakdown (Weber et al., 1993). However, this view changed after publication of data demonstrating that both fibroblasts and myofibroblasts have the capability of communicating (cell-to-cell) electrically and metabolically through conventional connexins within gap junctions. This connexin-mediated electrotonic communication may also include functional interactions of these fibroblasts/myofibroblasts with adjacent myocytes (Gaudesius et al., 2003; Chilton et al., 2007; Zlochiver et al., 2008; Kakkar and Lee, 2010). It is now apparent that both the fibroblast and the myofibroblast cell populations can serve as a significant source of paracrine substances that, when secreted, act on adjacent cells (Powell et al., 1999; Pedrotty et al., 2009; Rohr, 2011). Both fibroblasts and myofibroblasts can also be regulated by neurotransmitters, co-transmitters, peptides, and circulating hormones (Brilla et al., 1995; Rose et al., 2007; cf. Rose and Giles, 2008). Important early work on isolated fibroblasts also drew attention to the fact that the electrophysiological properties of these cells can be modulated significantly by mechanical perturbations, including both stretch and shear forces. Abramochkin et al. (2014) have recently reviewed this data.

Experimental work, based mainly on co-culture methods, combined with simulations using mathematical models of, e.g., human atrial myocytes and fibroblasts, have drawn attention to the possibility that the fibroblast cell population could significantly modulate the electrophysiological substrate in a number of cardiac tissues. Papers that deal specifically with these interactions in the atria include: (Maleckar et al., 2009a; Ashihara et al., 2012; McDowell et al., 2013) and ventricles (MacCannell et al., 2007; Sachse et al., 2008; McDowell et al., 2011) of the human heart. However, in all cases, these interactions have been assumed to be passive; that is, to not involve any regenerative or propagated responses that originate in the fibroblast/myofibroblast cell population (Gaudesius et al., 2003; Maleckar et al., 2009a; McDowell et al., 2011, 2013).

Important early work on atrial rhythm disturbances by Spach and his colleagues (Spach and Boineau, 1997) drew attention to the ability of “microfibrosis” to alter the electrical substrate in the atrium and thus promote arrhythmias. Spach et al. (2007) also recognized that in the human atrium an important consequence of aging was an increased incidence of supraventricular rhythm disturbances with some fibrosis. Enhanced fibrosis in the setting of healthy aging is now a well-known contributor to atrial flutter/fibrillation (Nattel, 2002; Biernacka and Frangogiannis, 2011; Diness et al., 2011; Guzadhur et al., 2012).

Recently, it has been reported that myofibroblasts derived from explanted human right atrial tissue (the right atrial appendage) can express measurable (and potentially functionally important) Na^+^currents. Specifically, Chatelier et al. (2012) have reported that approximately 75% of the myofibroblasts that are isolated from the human right atrial appendage express Na^+^ current after being maintained in a primary 2-D cell culture 8–12 days. They observed that 100% of these cells expressed Na^+^ current after 13–17 days in culture. These investigators have also provided evidence that this Na^+^ current is generated by the same Na^+^ channel α subunit, Na_v_ 1.5 that produces the Na^+^ current in the atria and ventricles of the adult human heart.

After the properties of this Na^+^-selective integral membrane protein in fibroblasts/myofibroblasts are understood more completely, a paradigm shift concerning the functional properties of human cardiac syncytium in both health and disease may be required. As an example, the expression of Na_v_ 1.5 in this cell population raises the possibility that the myofibroblast cell population could be the source of ectopic foci. That is, this Na^+^ current could provide a significant source of regenerative inward current. In principle, the resulting depolarization could serve both as the trigger and the substrate for supraventricular rhythm disturbances originating in the fibroblast/myofibroblast cell population.

The main focus of our study was to complement and extend the Chatelier et al. (2012) findings by addressing four separate, but related, objectives:

- To carry out whole-cell voltage clamp experiments and obtain the additional data that is needed for determining the probability that measurable Na^+^ currents, I_Na_, in fact can be recorded in single myofibroblasts from the human right atrial appendage, in situations where these cells are isolated, purified, and maintained in conventional 2-D culture.
- To further define the functional roles of I_Na_ based on its fundamental biophysical properties, e.g., voltage-dependence of activation and inactivation.
- To make PCR measurements aimed at identifying mammalian Na^+^ channel α and β subunits in this myofibroblast cell population.
- To integrate and illustrate the consequences of the Chatelier et al. (2012) findings and compare them with our results, through implementation of current mathematical models of the human atrial myocyte action potential coupled to a defined fibroblast/myofibroblast electrophysiological parameter set, and then to study these interactions *in silico* in selected ratios (myocytes/myofibroblasts) and coupling configurations.

Here, we report our preliminary findings based on conventional whole-cell voltage-clamp measurements of the electrophysiological properties of single myofibroblasts that were isolated from the human right atrial appendage and then placed in conventional 2-D tissue culture for approximately 6 days (2–4 passages). PCR analyses of the expression of a number of α and β subunits of mammalian Na^+^ channels are also presented. Our results are compared and contrasted with those in the original paper on this topic (Chatelier et al., 2012). Mathematical models of the human atrial myocyte and/or the fibroblast/myofibroblast are used to explore and illustrate plausible functional consequences of Na^+^ current expression in the myofibroblast population.

Taken together, this experimental and theoretical work contributes to ongoing discussions regarding possible functional roles for Na^+^ current in human atrial myofibroblasts. These findings also raise interesting possibilities and questions concerning novel molecular targets in the compromised human atrium, e.g., during persistent atrial fibrillation (Benito et al., 2008; Biernacka and Frangogiannis, 2011; Aguilar-Shardonofsky et al., 2012). This class of rhythm disturbance is of importance due to its increased incidence during the healthy aging process (Benito et al., 2008; Aguilar-Shardonofsky et al., 2012; Guzadhur et al., 2012); and because persistent atrial fibrillation can increase the incidence of transient ischemic attack and/or stroke (cf. Bersohn et al., 2012).

## Methods

### Human right atrial appendage tissue

Excised human atrial appendage specimens were obtained after written consent from patients undergoing coronary artery bypass graft surgeries at Foothills Medical Centre. Administrative approval was granted to the Fedak Laboratory by the University of Calgary Conjoint Health Ethics Board. After being transported to the Fedak Laboratory, each preparation was subjected to enzymatic treatment resulting in release of atrial fibroblasts, as described in previous papers from the Fedak Group (cf. Fedak et al., 2012).

### Cell culture methods

At selected times, (2 days to 2 weeks), populations of these myofibroblasts were transferred to the Giles Laboratory where they were maintained in conventional 2-D culture for an additional 1–2 days. As required, cells were released by enzymatic treatment (trypsinization) and then used for electrophysiological studies on that same day.

### Isolation of myofibroblast RNA and targeted PCR analyses

Messenger RNA was isolated from these cultured human atrial fibroblasts using a Qiagen RNeasy kit, and transformed into cDNA using Qiagen Quantitect reverse transcriptase kit according to the manufactures protocol. Real time-PCR analysis was performed using Quantitect SYBR Green Master Mix and primer pairs on a BioRad iCycler. Gene expression levels were calculated using the delta-delta CT method with 18s RNA serving as the housekeeping gene. Values represent the mean ± s.e.m. of three individual cell cultures performed in duplicate measurements. The primers for the analysis of human alpha and beta sodium channel subunits were generated at the University of Calgary DNA lab and BLASTed against the human genome to ensure 100% specificity against the targeted genes. To assess the possibility that expression of neonatal isoforms of the cardiac Na^+^ channel were upregulated, SCN5A, or Na_v_1.5, mRNA expression measurements were separated into primers recognizing splice variants 1 and 2, and primers recognizing variants 3 through 6 (see Table 1).

**Table 1:** **Primer sequences for PCR analyses of human atrial myofibroblast Na^+^ channel complex transcripts**.

**Channel**	**Gene**	**Forward**	**Reverse**
Na_V_ 1.1	SCN1A	ctccccacaccagtctttgt	tggtctgactcaggttgctg
Na_V_ 1.2	SCN2A	gccagcttatcaatcccaaa	tcttctgcaatgcgttgttc
Na_V_ 1.3	SCN3A	gtagtggtgcattggccttt	gcaccgagttctgagtagcc
Na_V_ 1.4	SCN4A	ttcacaggcatcttcacagc	ggaaggagcgtagcacagac
Na_V_ 1.5	SCN5A Var 1,2	cttcaccgccatttacacct	tgcgtaaggctgagacattg
Na_V_ 1.5	SCN5A Var 3,4,5,6	cttcaccgccatttacacct	aagttcgaagagccgacaaa
Na_V_ 1.6	SCN8A	tggacatcctttttgccttc	ctgcaggaccactgcagata
Na_V_ 1.7	SCN9A	ccacttcatccaccacctct	actgcactgccttcgagaat
Na_V_ 1.8	SCN10A	tctttgcagcttcttcagca	acccacacagtcggattagc
Na_V_ 1.9	SCN11A	cccagcagctgttaaaggag	ctgggacagtcgtttggttt
Na_V_β 1	SCN1B	tgcgctatgagaatgaggtg	atcttcttgacgacgctggt
Na_V_β 2	SCN2B	tgacccactctcttccatcc	caccaggtcctctctgaagc
Na_V_β 3	SCN3B	ccacacacccagacttcctt	tcctgcaaagatgcagtgac
Na_V_β 4	SCN4B	cccccacagtcttccaagta	gagagcagaggagcaggcta

### Electrophysiological methods

A small piece of coverslip with adherent cells was placed in a 200 μL recording chamber on the stage of an inverted microscope, where it was superfused with a HEPES-buffered solution (see below) at a rate of about 1 ml/min. The temperature was maintained at 20–22°C.

Membrane currents were measured in single human atrial myofibroblasts using whole-cell “patch clamp” techniques. Cells that were isolated from their neighbors, and lacked obvious connections to surrounding cells, were chosen for patch clamp recordings. Patch pipettes were pulled from borosilicate capillaries, and had a d.c. resistance of 4–9 MΩ when filled with pipette solution. Recordings of membrane current were made with a Multi-Clamp 700A amplifier (Molecular Devices; Sunnyvale, CA, USA). At least 75% series resistance compensation was used (series resistance <5 MΩ). When recording Na^+^ currents in the absence of K^+^ currents, a P/4 subtraction protocol was used to remove leak currents and reduce uncompensated capacity transient currents. Signals were digitized with a 1322A Digi-Data acquisition system (Molecular Devices), stored on a microcomputer and analyzed offline with pClamp v.8. Plots and statistical analysis were done with “Sigmaplot” (Systat Software; San Jose, CA, USA or “Prism” Graphpad Software, La Jolla, CA, USA).

**Figure 1:**
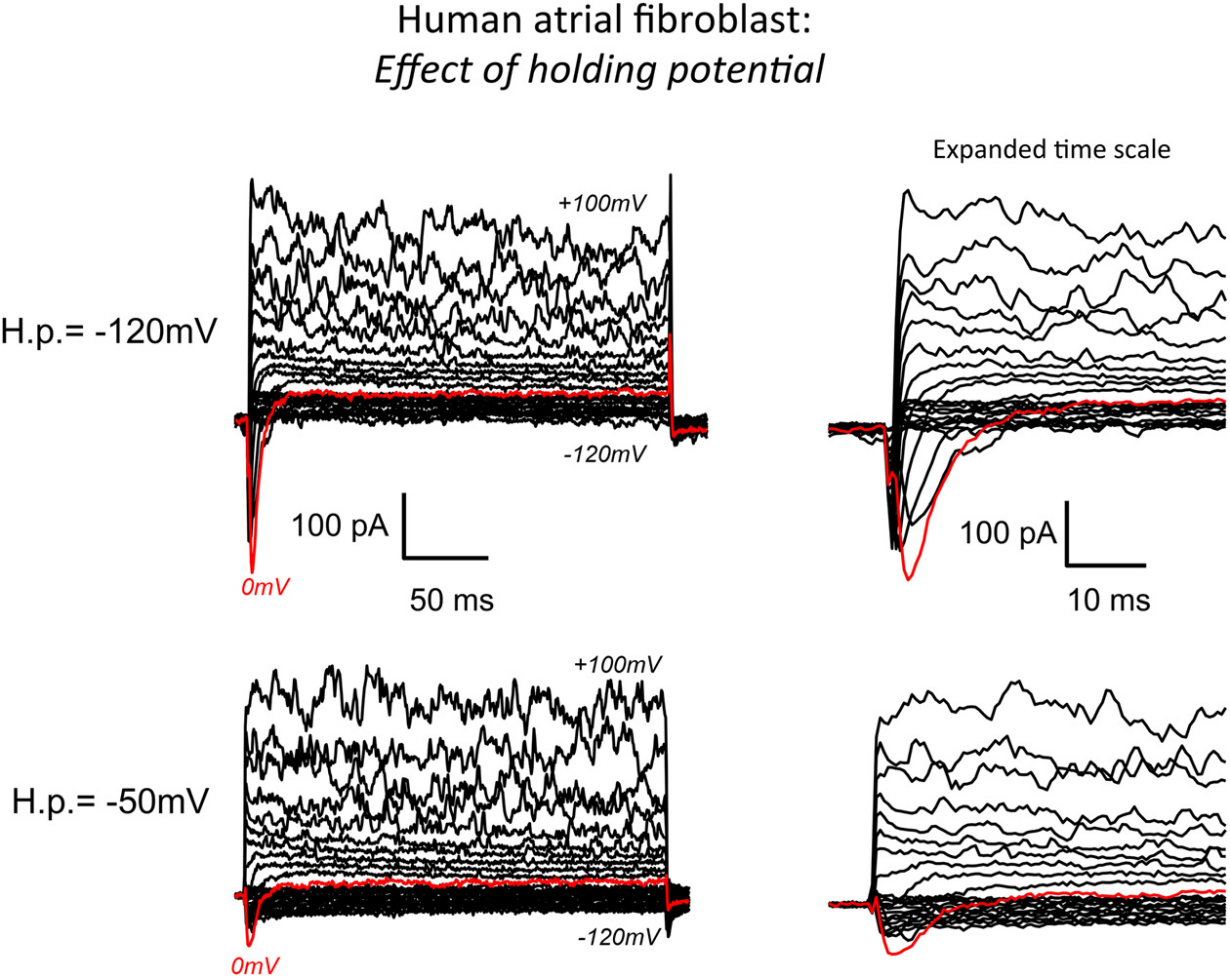
**Whole cell patch clamp records of the predominant transmembrane ionic currents recorded from an isolated human atrial myofibroblast using our standard superfusion solution at 22–23°C**. The pipette solution in this experiment was “K^+^-rich.” (See Methods for details of solutions and voltage-clamp protocols). The superimposed current records in the bottom left panel are typical of the data recorded when the voltage clamp “holding potential” (−50 mV) was set near the presumed resting membrane potential of the myofibroblast (approximately −40 mV). As shown, membrane depolarizations in the voltage range from about +20 to 100 mV resulted in a large “noisy” outward current that is likely generated by Ca^2+^-activated K^+^ channels. In this and most other recordings, hyperpolarization from HP −50 mV produced only very small current changes (see Chilton et al., 2007). Note, however, that depolarizing steps did elicit (red trace) a small but distinct transient inward current. The same records at an expanded time scale are shown on the right. The data in the top panel were obtained in response to the same levels of step depolarizations applied from a holding potential of −120 mV. As expected from the fundamental biophysical properties of a voltage-dependent Na^+^ (or Ca^2+^) current this hyperpolarized HP “unmasked” a much more prominent transient inward current (see Results).

**Figure 2:**
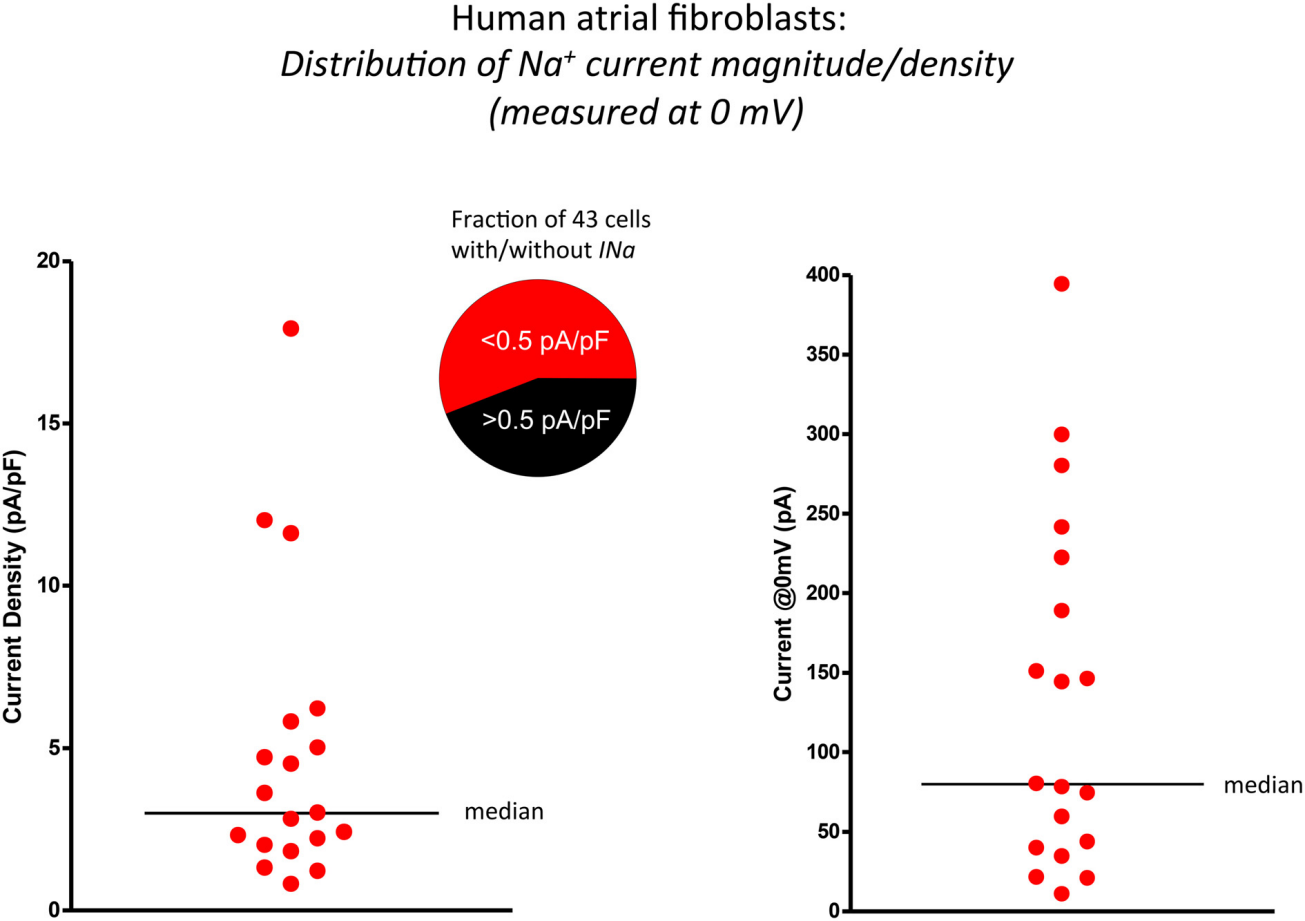
**Scatter plots illustrating the significant range of values of functional Na^+^ current expression in isolated human atrial myofibroblasts (*n* = 19 cells)**. The data in the left panel summarizes these findings as current densities, i.e., by expressing I_*Na*_ values (recorded in response to depolarization to 0 mV) normalized for cell (myofibroblast) capacitance (mean ± s.e.m.; 30.8 ± 4.9 pF). The same data set is presented in the right panel as peak I_Na_ at 0 mV. Horizontal bars in both plots indicate the median value of each distribution.

Current-voltage relations for the global transmembrane ionic current expressed in single human atrial myofibroblasts were obtained using a series of 200 ms voltage clamp steps from −120 to +100 mV (see Figure 1). For Na^+^ currents, a series of 100 ms steps from −50 to +40 mV (see Figure 2) were applied at 0.1 Hz. Steady-state inactivation of Na^+^ current was determined with a two-step protocol consisting of a 500 ms “conditioning” clamp step to −120 mV, followed by a “test” step to −10 mV. Cell capacitance was obtained from the displacement current transient produced by a +10 mV step from the holding potential (HP) −40 mV, which was close to the apparent resting membrane potential of these cells (see Discussion).

Analar grade chemicals (Sigma-Aldrich) were used to make all solutions. “Standard” external solution consisted of (mM): NaCl, 140; KCl, 5; CaCl_2_, 2; MgCl_2_, 1; HEPES, 10; glucose, 5.5; mannitol, 15. pH was adjusted to 7.4 with NaOH. K^+^-rich “internal” (pipette) solution consisted of (mM): K-aspartate, 100; KCl, 20; MgCl_2_, 1; Na2ATP, 4; CaCl_2_, 0.85; EGTA, 5; HEPES, 10. pH was adjusted to 7.2 with KOH. The approximate pCa of the solution was 7.9. Cs^+^-rich pipette solution, used to isolate Na^+^ currents, consisted of Cs-aspartate, 100; CsCl, 30; MgCl_2_, 1; Na2ATP, 4; CaCl_2_, 0.85; EGTA, 5; HEPES, 10. pH was adjusted to 7.2 with CsOH.

### Mathematical modeling of the human atrial action potential and myofibroblast/myocyte interactions

Mathematical modeling was done using a combination of (i) the Koivumäki et al. model (2011) of the human atrial action potential and intracellular [Ca^2+^]_i_ changes, and (ii) the “active” model of the human atrial myofibroblast described in detail in Maleckar et al. (2009a,b). For our simulations, the mathematical expression for the inwardly rectifying background K^+^ current, I_K1_, in the fibroblast originally developed by MacCannell et al. (2007) was modified. This was done so that this important non-linear background current, and its contribution to the fibroblast resting potential could be simulated more accurately. Specifically, the equation for I_K1_ in Nygren et al. (Nygren and Giles, 2000) was used, and I_K1_ current density was scaled for the size (capacitance) of the fibroblast. This adjustment required a directly related small change in the size of the Na^+^/K^+^ pump current.

**Figure 3:**
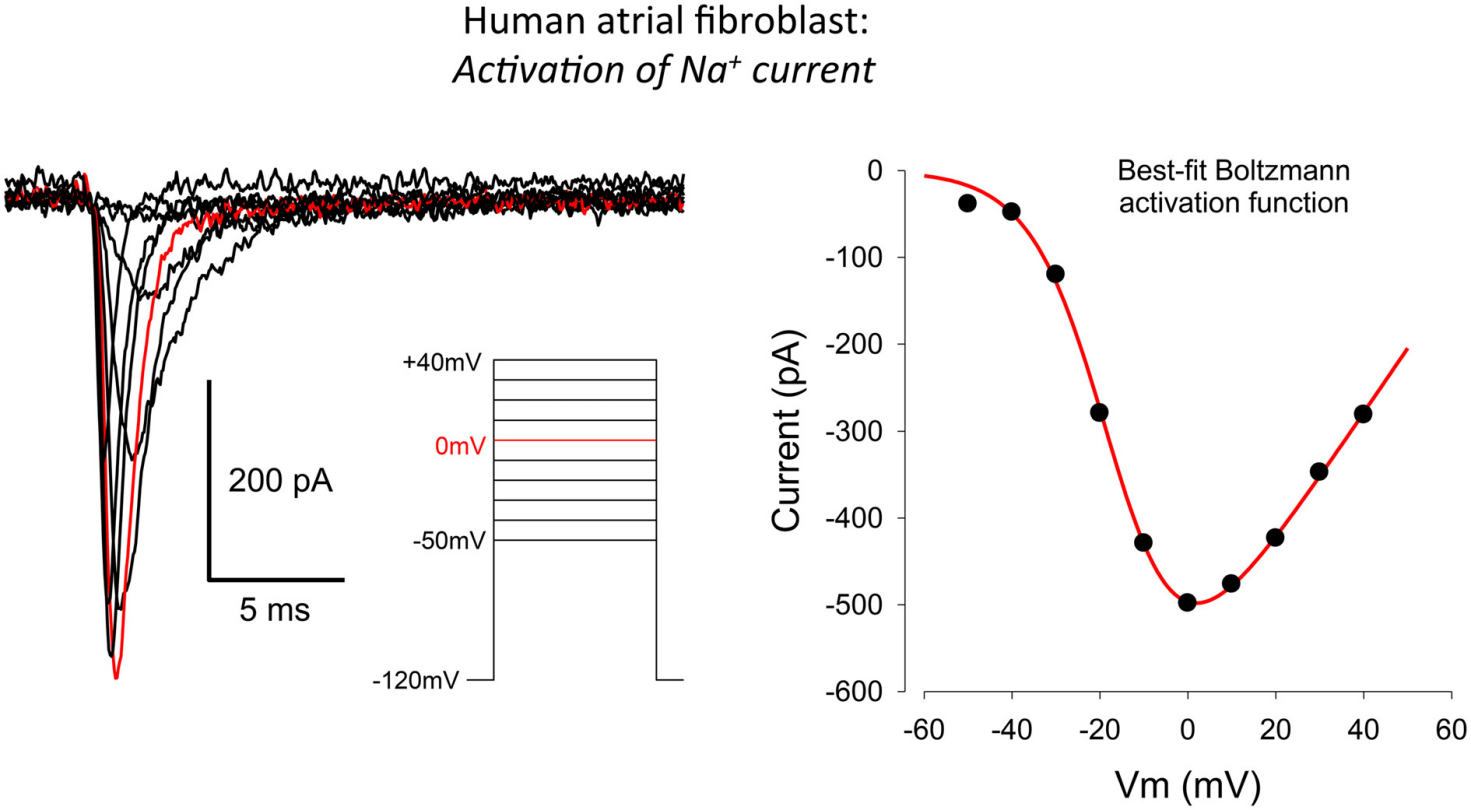
**Whole-cell voltage clamp records illustrating the activation of the transient inward Na^+^ current in human atrial myofibroblasts**. Raw data (10 superimposed current records) elicited by 100 ms depolarizing clamp steps from −50 to +40 mV are shown; the red trace indicates the current elicited at 0 mV. The voltage clamp protocol is shown in the inset. The plot in the right-hand panel is an I–V curve. It was constructed by plotting the peak inward current at each selected voltage clamp depolarization level. The patch pipette solution in this experiment was Cs^+^-rich (Methods), to block the large and rapidly activating I_K-Ca_, which obscured I_Na_ at potentials positive to about +30 mV (see Figure 1 and text in Results).

**Figure 4:**
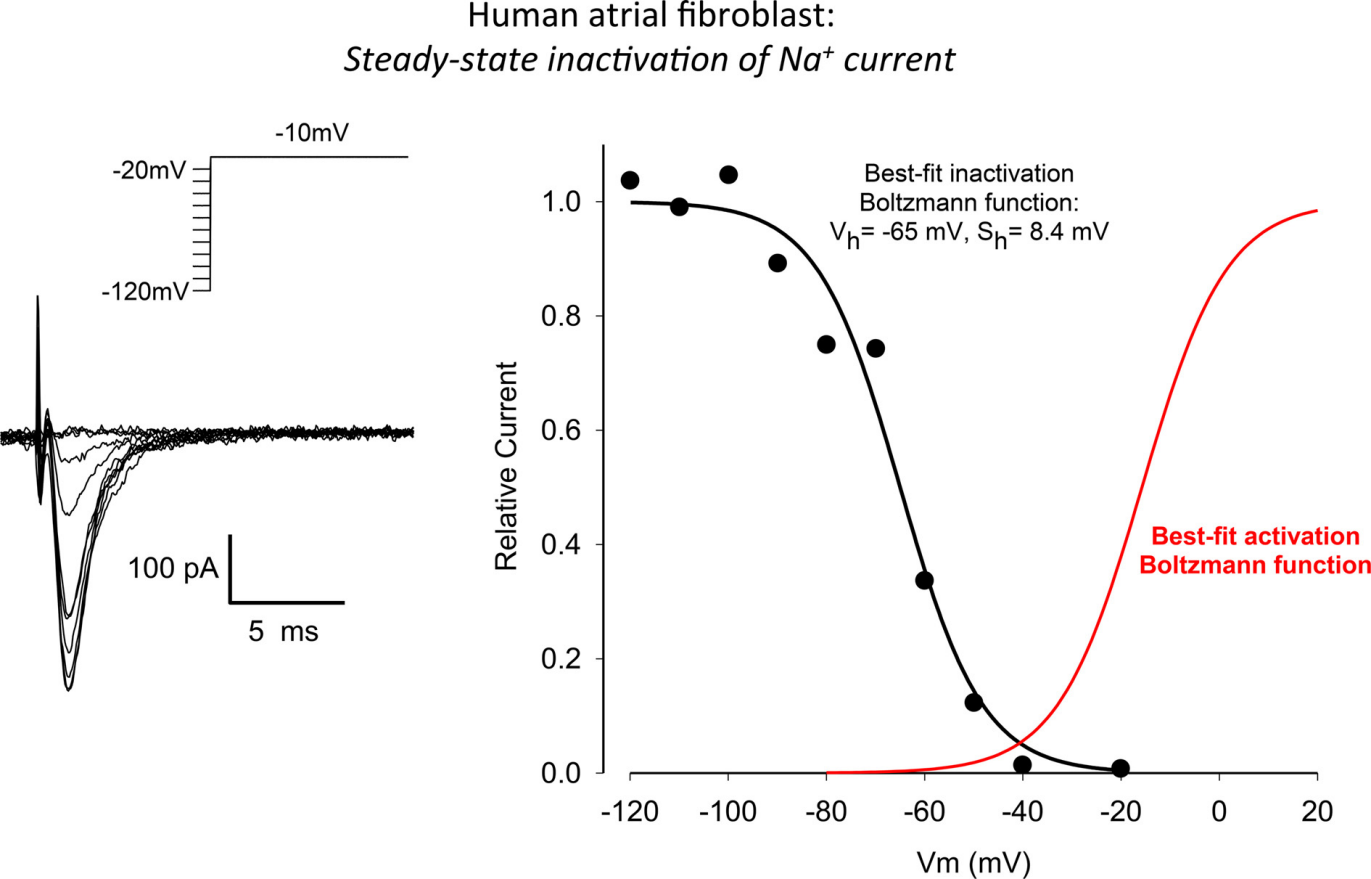
**Steady-state voltage-dependence of inactivation of I_Na_ in a human atrial myofibroblast (same cell as in Figure 3)**. The superimposed current records in the left panel were obtained in response to the conventional two-step voltage clamp protocol that is illustrated in the inset (500 ms “conditioning” step from −120 mV, “test” step to −20 mV). Peak inward I_Na_ values are plotted in the right-hand panel (continuous black curve) vs. the conditioning or prepulse potential. These results reveal a conventional inactivation relationship for I_Na_ in an adult mammalian myocyte with the half-inactivation membrane potential and maximum slope at V_1/2_ being −65 and 8.4 mV, respectively. The relationship shown in red—a derived voltage-dependent activation curve—is based on the data in Figure 2. See Results for further, more detailed description.

Three variants of this myofibroblast/atrial myocyte model were used in the simulations shown in **Figures 6**–**8**:

- V_o_: the original model developed by Maleckar et al. (2009a) with I_K1_ modified as described.
- V_1_: V_o_ with the addition of a Na^+^ current based on the electrophysiological data shown in Figures 1–**4**.
- V_2_: V_o_ after Na^+^ current was parameterized based on the *in vitro* data published by Chatelier et al. (2012), as opposed to our data.

The illustrations in **Figures 6**–**9** were generated by using these myocyte/myofibroblast models, assuming connexin-mediated electrotonic cell-to-cell communication with an assumed gap junctional conductance of 0.5 nS. This value corresponds to the lower end of those measured between fibroblast-myocyte pairs *in vitro* (Maleckar et al., 2009a,b).

In addition to a 1:1 myocyte-myofibroblast coupling scheme, two slightly more complex sets of starting conditions were explored in these simulations. Either 3 or 9 myofibroblasts were connected in series to 1 myocyte. Based on our findings that only approximately 30–50% of the isolated myofibroblasts express a measurable Na^+^ current, only approximately one third of these myofibroblasts models were programmed to express a Na^+^ current with the properties in Figures 1–**4** (model variant V_1_).

## Results

A conventional whole-cell patch clamp method was used to characterize the predominant transmembrane ionic currents in these single isolated human atrial myofibroblasts. Representative families of membrane currents are shown in Figure 1. In this myofibroblast and in the majority of others that were studied, voltage clamp depolarizations resulted in rapid activation of a small outward current. Larger depolarizations (in the +60 to +100 mV range) consistently gave rise to large “noisy” transmembrane records, that are characteristic of Ca^2+^-activated K^+^ currents. In contrast, hyperpolarizing clamp steps from the holding potential produced much smaller changes in transmembrane current, similar to the pattern that has been reported in rat ventricular fibroblasts/myofibroblasts (Chilton et al., 2005).

A main focus of this study was to determine whether transient inward Na^+^ currents could be detected in these myofibroblasts. For this reason, families of transmembrane currents were generated from two different holding potentials. After each cell was voltage clamped near the resting potential, the holding potential was set to −50 mV, and step depolarizations lasting 100 ms were applied in 10 mV increments covering the range of membrane potentials −120 mV to +100 mV. As shown in the bottom row of Figure 1, this protocol produced small but measurable transient inward currents (indicated by the red traces). To enhance the likelihood of detecting Na^+^ current, and to attempt to record the maximum transient inward Na^+^ current that could be generated by each individual myofibroblast, the holding potential was then set to −120 mV. Transmembrane currents, in response to the same levels of depolarization, were elicited. These families of currents are shown in the top row of Figure 1. As expected (as a consequence of the more hyperpolarized holding potential removing inactivation), this maneuver resulted in significantly larger transient inward currents (see red traces). This type of protocol was applied to 40 myofibroblasts in total: the data indicate that approximately 50% of these isolated myofibroblasts express a measurable transient inward current, when the holding potential was −120 mV (see Discussion).

In this series of experiments, it became apparent that there was considerable variability in the expression levels of these Na^+^ currents. The scatterplots in Figure 2 illustrate and summarize this variability. Note that when the transient inward Na^+^ current is measured, either in terms of current density (left) in response to a depolarization to 0 mV or plotted as peak current, (right) there is very significant heterogeneity or scatter. The average current density at 0 mV was approximately 3 pA/pF when the holding potential was −120 mV.

The raw data (left) and current-voltage relationship (right) in Figure 3 provide useful information concerning the peak size, as well as the activation and inactivation kinetics, and the voltage-dependence of this peak inward current in the human atrial myofibroblast, respectively. The family of currents shown in the left panel of Figure 3 was obtained by applying rectangular voltage clamp depolarizations from a holding potential of −120 mV, as illustrated in the inset. The peak current in response to this step-depolarization to 0 mV is highlighted in red. This current trace provides a point of reference for the summarized data described in Figure 2.

A representative peak inward current-voltage relationship is shown in the right panel of Figure 3. Note that this inward current first activates at a membrane potential near −50 mV, and that it is maximal near 0 mV. The positive limb of this I–V curve could not be obtained in its entirety due to the rapid activation of a substantial and noisy outward current (see Figure 1) at membrane potentials positive to approximately +50 mV. Overall, this pattern of current changes closely resembles families of currents and peak current-voltage relationships that have been published for Na^+^ current in cardiac myocytes. This includes myocytes from human atrium (Baba et al., 2006), and data based on heterologous expression, when the α subunit of the cardiac Na^+^ channel, Na_v_ 1.5 is overexpressed in mammalian cell lines, and then studied with conventional patch clamp methods.

The raw data and corresponding analysis shown in Figure 4 summarize our experimental measurements of the steady-state inactivation characteristics of the Na^+^ current in these human atrial myofibroblasts. A conventional two-step voltage clamp protocol was used to progressively inactivate this Na^+^ current. From a holding potential of −120 mV, a relatively long “inactivating” prepulse (500 ms) was applied, and this was followed immediately by a second step-depolarization to −10 mV. As expected and as illustrated in the superimposed currents in the left panel, when the prepulse voltage depolarized the cell beyond approximately −40 mV, peak Na^+^ current decreased progressively. This voltage-dependent inactivation was characterized over the entire physiological range of membrane potentials, as shown in the plot in the right panel of this Figure. The best-fit parameters for this steady-state inactivation relationship were: membrane potential for half an activation or V_h_ −65 mV, and maximum slope factor 8.4 mV. In this panel of Figure 4, the sigmoid relationship shown in red is a derived relationship depicting voltage-dependent activation for the Na^+^ currents shown in Figure 3 (right).

These electrophysiological findings are somewhat similar to those in the original paper on this topic (Chatelier et al., 2012). Certainly, both data sets reveal the functional expression of Na^+^ channels in tissue cultured human atrial myofibroblasts.

**Figure 5:**
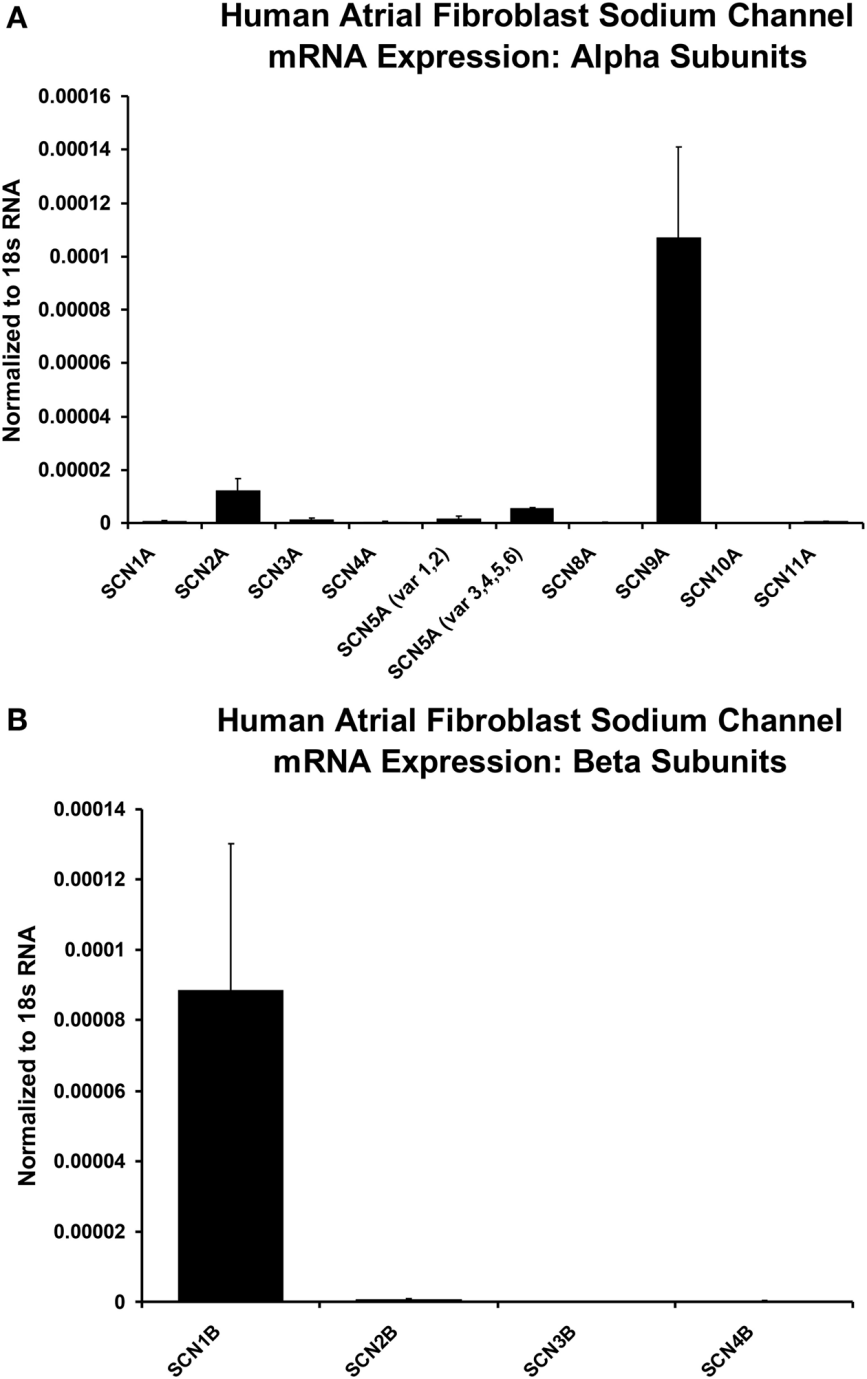
**Histograms of normalized data from PCR measurements of relative levels of Na^+^ channel α and β subunits, based on RNA derived from populations of isolated human atrial myofibroblasts maintained in 2-D tissue culture**. Note from panel **(A)** the predominant α subunits are SCN9A and SCN2A; Panel **(B)** shows that the strongest β subunit signal is for β_1_. However, the cardiac isoform of Na^+^ current α subunit, SCN5A, also can be detected (see Discussion). Sample size for mRNA measurements are from *n* = 3 individual samples for plated and collagen treated fbs (only plated values are shown). The statistical analysis was based on the difference between plated and collagen treated cells, with a simple unpaired *t*-test to compare gene expression under the two conditions.

Additional molecular evidence for the expression of Na^+^ channels in these human atrial myofibroblasts is presented in Figure 5. These histograms are based on our PCR analyses of the relative expression levels of a range of human Na^+^ channel (SCN-5) α and β subunits. From panel A note the two strongest α subunit signals are for the “nerve” Na^+^ channels SCN9A and SCN2A, respectively. However, the predominant (TTX-insensitive) mammalian heart α subunit was also be detected. Figure 5B presents analogous PCR data directed toward Na^+^ channel β subunits. Here, the β_1_ subunit represents the predominant signal (see Discussion).

**Figure 6:**
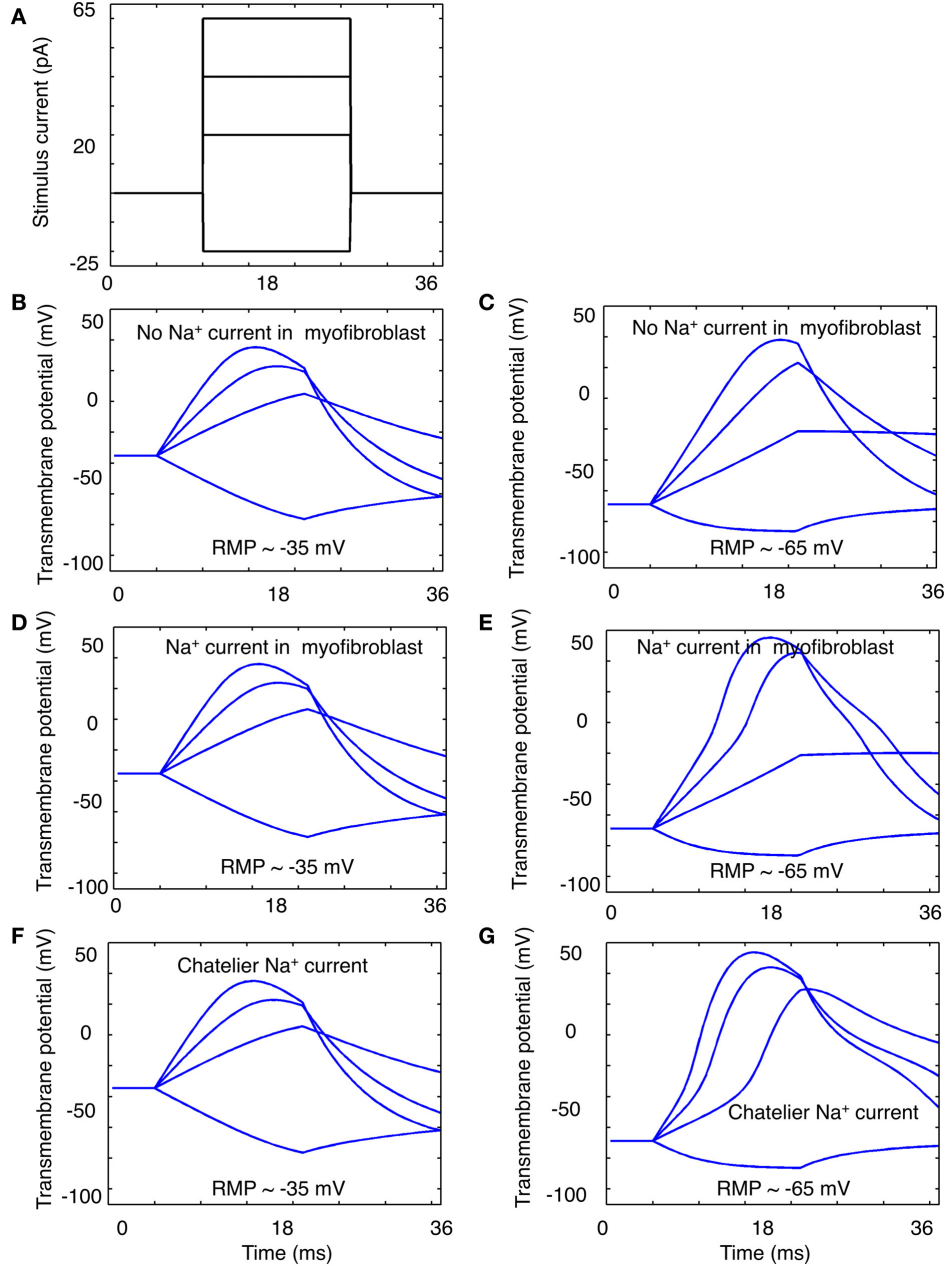
**Illustration of “electrotonic” responses in an *in silico* human atrial myofibroblast produced by 16 ms constant current stimuli that progressively depolarize or hyperpolarize the myofibroblast model**. The stimulus protocol is shown in panel **(A)**. The superimposed responses in **(B,C)** were obtained using a modified (see Methods) MacCannell et al. (2007) model of an atrial myofibroblast with no Na^+^ current added. The families of responses in **(D,E)** were obtained when this model also included equations for the Na^+^ current in this paper; or the Na^+^ current in the Chatelier et al. paper (2012) **(F,G)**. Note when Na^+^ current is included, most of the depolarizing stimuli produce a biphasic response. This may suggest a contribution of Na^+^ current activation to the stimulus-induced depolarization (see Discussion).

**Figure 7:**
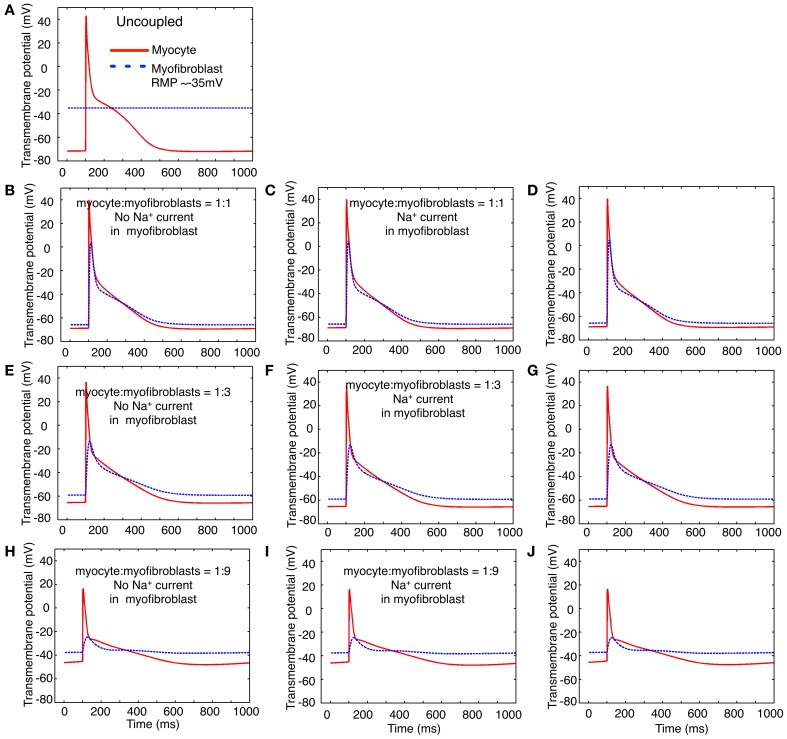
**Illustration of the effects of coupling selected numbers of myofibroblasts to 1 human atrial myocyte using an *in silico* hybrid mathematical model similar to the one first described by Maleckar et al. (2009a) when assuming a myofibroblast resting potential of −35 or −65 mV**. Panel **(A)** shows the human atrial myocyte action potential (red) and the myofibroblast at a resting potential of −35 mV, prior to these cells being coupled through a linear intercellular conductance of 0.5 nS. The remainder of this Figure is arranged in three rows. Panels **(B–D)** illustrate responses obtained when the myocyte to myofibroblast ratio is 1:1, panels **(E–G)** were computed based on a myocyte to myofibroblast ratio of 1:3; and panels **(H–J)** show responses when myocyte to myofibroblast ratio was 1:9. The arrangement of the vertical columns allow a direct comparison of the electrophysiological effects of (a) there being no Na^+^ current in any myofibroblast (left column, panels **B,E,H**); (b) the Na^+^ current identified in our laboratory and described in the paper being introduced (panels **C,F,I**); or (c) the Na^+^ current reported by Chatelier et al. (2012) being added to each myofibroblast **(D,G,J)**. Note that the myocyte action potential causes a significant electrotonic waveform in each myofibroblast at ratios of 1:1 and 1:3. At a coupling ratio of 1:9, the myofibroblasts exert a pronounced depolarizing influence of the myocyte resting potential in these assumed conditions (see Discussion).

**Figure 8:**
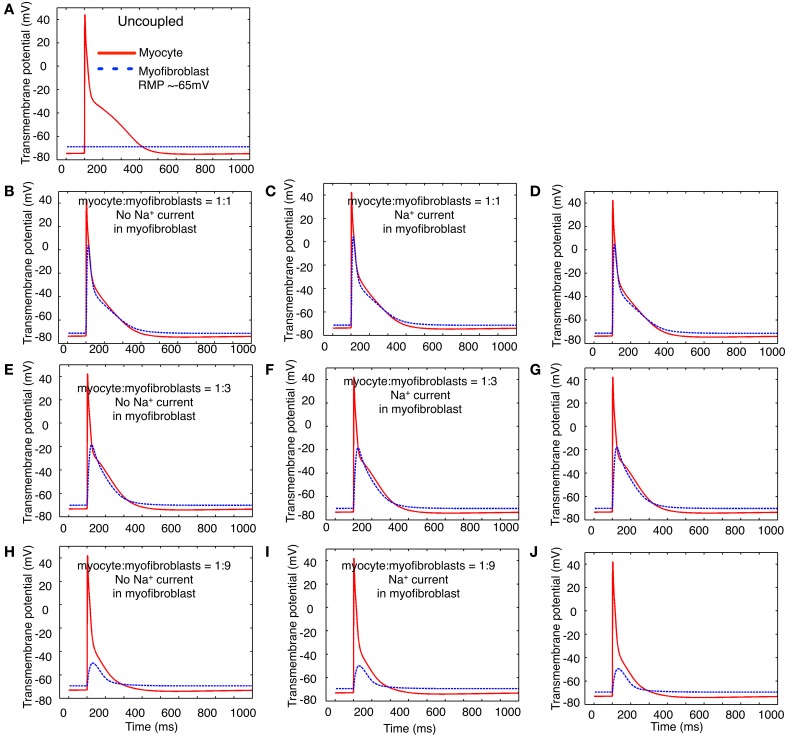
**Illustration of the effects of coupling selected numbers of myofibroblasts to 1 human atrial myocyte using an *in silico* hybrid model similar to the one first described by Maleckar et al. (2009a), assuming a myofibroblast resting potential of −65 mV**. The layout of this Figure is the same as that of Figure 7. Note that two potentially important differences in the patterns of *in silico* responses were obtained: (1) in all situations, the myofibroblast electrotonic responses are larger, (2) the myofibroblasts exert only a minimal depolarizing influence on the resting potential of the atrial myocyte.

Our data (Figures 1–5) and the Chatelier et al. publication (2012) that first reported functional expression of Na^+^ current in human atrial myofibroblasts suggest the possibility that these cells may be able to exhibit regenerative, action potential-like responses when appropriate depolarizing stimuli are applied. Figures 6–**8** illustrate the results of *in silico* tests of this possibility.

For these simulations, two quite different starting conditions were chosen: (i) the myofibroblast was assumed to have a resting membrane potential E_m_ of −35 mV, and (ii) the myofibroblast E_m_ values were set at −60 mV. This was done in recognition of the uncertainty of the fibroblast resting potential *in situ* (see Discussion), and because the Na^+^ current is inactivated almost completely at −35 mV. In both cases, selected 16 ms de- and hyperpolarizing stimuli were applied intracellularly, as diagrammed in Figure 6A.

The results in Figures 6B,C illustrate the responses when the myofibroblast was stimulated from a steady membrane potential of −35 mV, and no I_Na_ was introduced/expressed. As expected, the myofibroblast membrane potential responds to small rectangular stimuli with a quasi-exponential electrotonic de- or hyperpolarization, having characteristic time-courses governed approximately by the product of the mean input resistance (3–5 Gigohms) and single cell capacitance (6–10 pF). Note that the original mathematical model of the myofibroblast employed in these simulations (MacCannell et al., 2007) includes a rapidly activating delayed rectifier K^+^ current, in accordance with experimental data from rat ventricular myofibroblasts. This K^+^ current is activated when E_m_ depolarizes positive to approximately −30 mV. Indeed, the myofibroblast exhibits a partial repolarization (top trace, panel B) at positive membrane voltages (approximately 0 mV). In addition, a post-stimulus hyperpolarization due to the subsequent deactivation of this K^+^ current was observed. The results in Figure 6D,E were obtained using the same 4 constant current stimuli (panel A) after the Na^+^ current (identified in our study; Figures 1–4) was introduced. Note (panel **E**) that there is a tendency for regenerative depolarization in the voltage responses corresponding to all 3 depolarizing stimuli when E_m_ is set at −65 mV. The data in panels **F** and **G** is analogous to that in panels **D** and **E**, except that in these simulations I_Na_ equations that replicate the Na^+^ current described by Chatelier et al. (2012) have been “introduced” into the myofibroblast.

In the next two sets of computational studies, a mathematical model of the human atrial myocyte was combined with the modified mammalian myofibroblast model that is described in Methods. Three sets of computations were done: (i) a fixed number of *in silico* myofibroblasts were coupled to a single human atrial myocyte through a linear resistance corresponding to a conductance of 0.5 nS. These results are shown in the left column, panels **B**,**E**,**H** of Figure 7. (ii) In the second set of computations, Na^+^ current consistent with the biophysical properties depicted in Figures 1–4 in this paper was introduced into the myofibroblast cell population. These results are shown in the middle column, or panels **C**, **F**, and **I** of Figure 7. (iii) Finally, an analogous set of computations was done, this time using the Na^+^ current having the biophysical properties reported by Chatelier et al. (2012). In Figure 7, these computations are shown in the third column, or panels **D**,**G**,**J**. For a reference and starting point, panel **A** shows the atrial myocyte resting potential and action potential waveform (red); and also illustrates the assigned myofibroblast resting potential when these two types of cells are *not* coupled. Overall, this pattern of responses was *not* particularly revealing with respect to Na^+^-current dependent patterns of action potential waveforms or the electrotonic responses in the myofibroblast population. Specifically, the expression of Na^+^ current in the myofibroblast appeared to have *no* significant effects on the electrotonic waveform.

Note, however, that an important variable was that the myofibroblast resting membrane potential was initially set to that reported for these isolated cells, namely approximately −35 mV. After coupling in the 1:1 (panels **B–D**) and 1:3 (panels **E–G**) cases the fibroblast hyperpolarized and experienced a significant electrotonic depolarization and repolarization driven by the myocyte action potential. In the case of 1:9 ratio coupling (panels **H–J**) the atrial myocyte depolarized significantly and exhibited only a weak regenerative response, which resulted in a small electrotonic depolarization in the myofibroblast(s). We note that when the resting potential in either the myocyte or the fibroblasts is positive to approximately −60 mV, I_Na_ would be strongly inactivated (see Figure 4), and thus would not contribute to the membrane voltage profile in the myofibroblast or the immediately adjacent myocyte.

After recognizing that the resting membrane potential of the myofibroblast was a critically important determinant of the size of the peak inward Na^+^ current, a second set of computations was carried out with the membrane potential of the myofibroblast set to approximately −65 mV, rather than −35 mV. In Figure 8, the presentation of the data is the same as that of data Figure 7. Thus, the panels in column 1 (**B**,**D**,**E**,**H**) were based on computations done with no Na^+^ current in the myofibroblast cell population, and with coupling ratios of myocyte to myofibroblasts of 1:1, 1:3, and 1:9, respectively. As expected from our previous papers (MacCannell et al., 2007; Maleckar et al., 2009a), the myocyte membrane potential dominates that of the myofibroblast at low myofibroblast/myocyte ratios. In distinction, to when the myofibroblast resting membrane potential is much more depolarized (−35 mV), when the coupling ratio is 1:3 or 1:9, both the myofibroblasts and the myocytes have well-polarized resting membrane potentials. Partly for this reason, the waveform of the voltage change in the myofibroblast also differs substantially. This arises from the fact that more hyperpolarized resting membrane potential removes inactivation from the Na^+^ channels. This effect would be expected to be strongly manifested in the computations shown in panels **C**,**F**,**I**—as well a **D**,**G**,**I**, since the voltage dependence for inactivation of the Na^+^ current falls well within the range of the resting membrane potential of the myofibroblast.

Careful inspection of some of the computational results in Figures 7, 8 may suggest that activation of I_Na_ in the myofibroblast influences the overall “substrate” electrophysiological profile. Thus, in some cases, the membrane potential in the myofibroblast(s) depolarizes, when the atrial myocyte is repolarizing.

**Figure 9:**
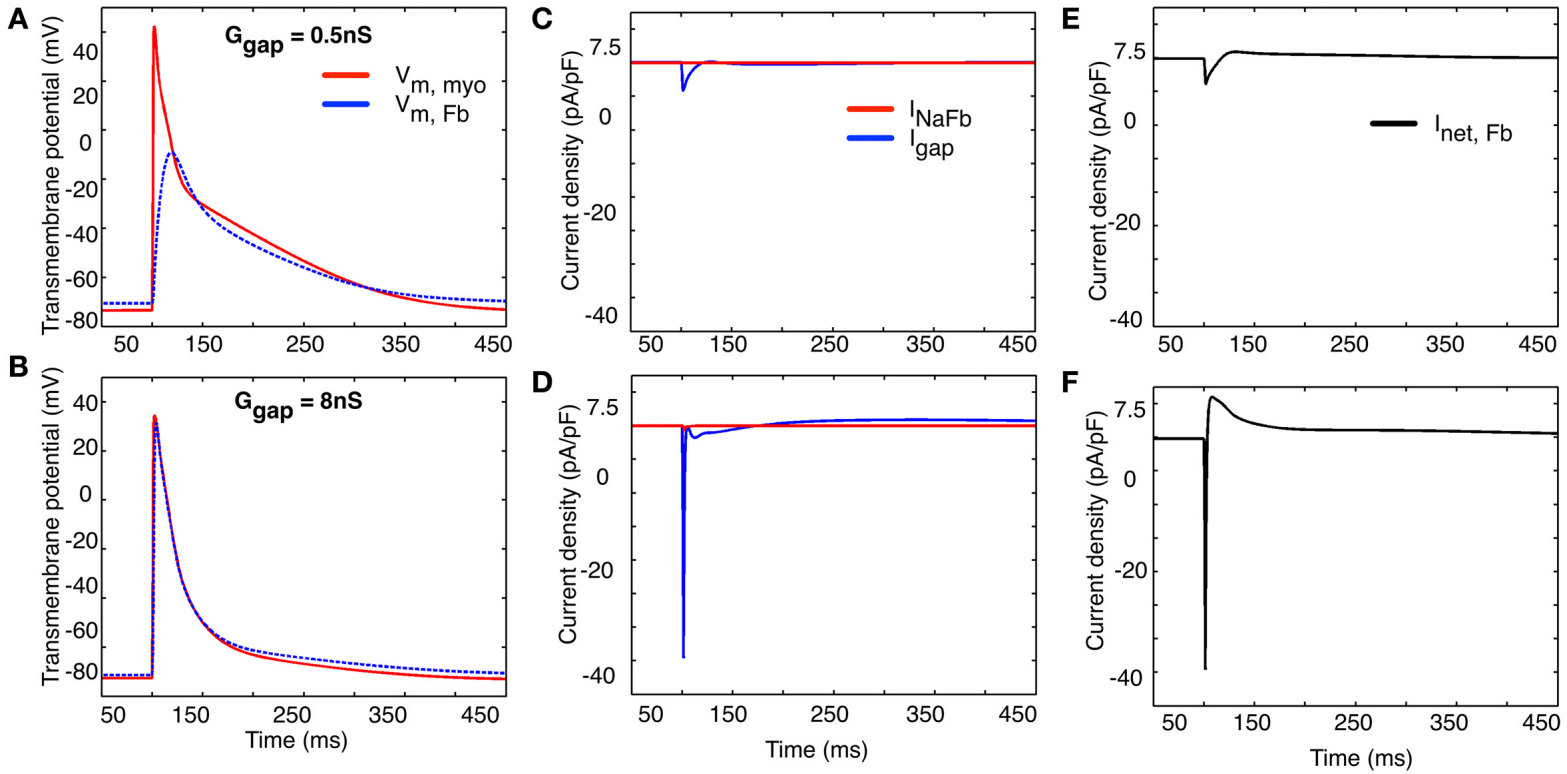
**Illustration of two selected values of the effects of coupling resistance on the electrotonic membrane potential (panels A,B), “gap current” and current flow between the two cell populations (1 myocyte: 1 myofibroblast, panels C,D) and the net transmembrane current in the collective myofibroblast population (panels E,F)**. See text in Results for further explanation.

The computational results shown in Figure 9 provide some further insight into this intriguing possibility. All of these computations were done using a 1 myocyte to 1 myofibroblast ratio. Two different values of coupling (intercellular) resistance, either 0.5 nS (top row) or 8 nS (bottom row) were selected for study. As expected, when intercellular resistance is very small, the myocyte action potential produces an electrotonic response in the myofibroblast that is essentially unattenuated.

Note that at both these values of intercellular resistance (panels **C**,**D**), the current flowing between the “cells” is dominated by the net inward (depolarizing) and outward (repolarizing) currents generated by the *atrial* myocyte. Panels **E** and **F** show the net transmembrane current in the myofibroblast population. Again, the inward, depolarizing current appears to be contributed mainly by the activation of I_Na_ in the *atrial* myocyte(s), as opposed to the myofibroblast(s) (see Discussion).

## Discussion

### Main findings

The whole-cell voltage clamp measurements shown in Figures 1–4} confirm that when human atrial fibroblasts are isolated and then placed in conventional 2-D culture, a sizeable fraction (approximately 50%) of these cells (myofibroblasts) express a measurable Na^+^ current. A number of electrophysiological and biophysical features of this current are noteworthy. (i) Its peak amplitude (in the range: 0.8–17.9 pA/pF) is sufficient to suggest that the myofibroblast may exhibit a regenerative action potential. (ii) The steady-state voltage dependence of inactivation, and the derived relationship that depicts quasi steady-state voltage dependence of activation, both strongly resemble the analogous biophysical parameters for the Na^+^ current recorded in adult human atrial myocytes (Baba et al., 2006). (iii) These findings, in conjunction with the PCR data in Figure 5, provide initial evidence that this current is similar to the Na^+^ current in an adult human atrial myocyte, as was originally reported by Chatelier et al. (2012). However, we note that our PCR analysis yielded data that identifies two nerve Na^+^ channel α subunits, Na_v_1.9 and Na_v_1.2, as the predominant transcripts. The cardiac Na^+^ channel α subunit was detectable in our myofibroblast preparation, but was less prominent than either Na_v_1.9 or Na_v_1.2.

Some of our findings differ from those in the original paper (Chatelier et al., 2012) that first identified Na^+^ current in human atrial myofibroblasts. One of their most striking findings was that this Na^+^ current exhibited a very hyperpolarized range of membrane potentials for activation and inactivation (see Figures 2B, 3A, in Chatelier et al., 2012). The reasons for these important differences may be due to the fact that some of the experimental conditions in the two studies differ. In the Chatelier et al. work (2012), the pH of the “internal solution” was 7.4; and perhaps more importantly, CsF rather than CsCl was used in the pipette-filling solution. It is well-known that F^−^ in the internal solution can cause a pronounced hyperpolarizing shift in Na^+^ current gating parameters (Chandler and Meves, 1970). An important consequence of the gating variables for the Na^+^ current being shifted in the hyperpolarizing direction in the Chatelier et al. paper (2012) is that the myofibroblast resting potential would need to be negative to −60 mV before this current could be activated. Second, in principle, there could be a substantial non-inactivating or “background/steady” Na^+^ influx, when the myofibroblast membrane potential is in the voltage range approximately −90 to −40 mV. This would depolarize the myofibroblast and tend to increase [Na^+^]_i_ and have significant secondary consequences on [Ca^2+^]_i_, and [pH]_i_ homeostasis.

These considerations raise the very important question: what is the resting potential of the human atrial fibroblast/myofibroblast? Values in the range −30 to −50 mV are often reported for isolated single cell mammalian fibroblast/myofibroblast preparations. However, accurate measurement of membrane potential in these small cells is very challenging (Dubois, 2000), especially under conditions, in which a background inwardly rectifying K^+^ current is expressed (Wilson et al., 2011). This is the case in cardiac fibroblasts/myofibroblasts (Chilton et al., 2007), since an inwardly rectifying background current is expressed. It is also useful to recall that, as it is in fibroblasts, the classical experiments of the Kiseleva Group (Kiseleva et al., 1998; Kamkin et al., 2002; cf. Kohl, 2003) show that the mechano-sensitivity of cardiac fibroblasts can significantly change membrane potential (Abramochkin et al., 2014). It is likely that fibroblast membrane potential may vary with the mechanoelectric activity of the heart.

As mentioned, the data from our PCR measurements differ from the original findings (Chatelier et al., 2012) concerning the molecular identity of the Na^+^ current α subunit(s) in human atrial myofibroblasts (see Figure 6). Thus, we suggest that the major α subunits are Na_v_1.9 and Na_v_ 1.2, as opposed to Na_v_ 1.5. Of the four known Na^+^ channel β subunits that could, in principle, contribute to the Na^+^ channel complex (Abriel and Kass, 2005), the β_1_ subunit predominates in the human atrial myofibroblasts in the mammalian heart, as is the case in the corresponding adult myocyte. At present, we cannot explain the significant difference in the pattern of Na^+^ channel α subunit expression. However, it is possible that our cell culture preparation is less uniform than that of Chatelier et al. (2012). Our PCR analysis was done using a preparation that had been plated on coverslips only 24–48 h before being studied. However, these myofibroblasts had been passaged a number of times (2–6) before they were “released and plated.” In contrast, Chatelier et al. studied myofibroblasts after some 3 weeks in culture, corresponding to approximately passage 9. This difference is an important issue that can only be resolved by a separate, much more extensive study.

Our study did not focus on the molecular pharmacology of the Na^+^ currents in the myofibroblast. However, the original paper (Chatelier et al., 2012) reported (Figure 5) that tetrodotoxin (TTX) did block this current. In these experiments, the effective concentrations of TTX were in the 10–100 μM range. This further supports its identity as a cardiac myocyte-like Na^+^ current, generated by the complex of Na_v_ 1.5 and the β_1_ subunit.

### Computational work

It is somewhat surprising that introduction of Na^+^ current into the modified MacCannell model of the mammalian fibroblast (MacCannell et al., 2007) did *not* result in any convincing action potential-like regenerative responses (Figures 6, 7). With respect to the results shown in Figure 6, this may be due to the fact that the relatively depolarized “assigned” resting potential (−35 or −65 mV) of the fibroblasts/myofibroblasts resulted in very significant inactivation of I_Na_. A second contributing factor may be the incomplete information concerning the kinetics of activation and inactivation of I_Na_ in the human atrial myofibroblast. The high input resistance of these cells (5–10 Gigohms) results in a large intrinsic membrane time constant; and this may contribute to Na^+^ current “accommodation”—inactivation developing at a rate similar to activation at membrane potential near the normal firing threshold for I_Na_.

At this stage of its development, the hybrid modeling illustrated in Figures 7, 8 does not independently reveal significant new information. Perhaps the most realistic myocyte to myofibroblast ratios that we have explored is the 1:3 case. In this defined starting condition, we confirm that the myocyte action potential produces a significant electrotonic waveform in the connexin-coupled myofibroblasts (MacCannell et al., 2007; Maleckar et al., 2009a). In addition, when large differences in the resting potential of the myofibroblast vs. the myocyte are assumed/assigned, the myofibroblast can significantly depolarize the atrial myocyte. This depolarization would be expected to reduce myocyte excitability and conduction velocity; and may significantly change diastolic Ca^2+^ levels. It is worth noting that these effects could be anticipated to be spatially heterogeneous within the human atrial substrate.

### Translational perspectives

The expression of Na^+^ current (more specifically the detection of essential components of the Na^+^ channel complex) in the myofibroblast, may be important for reasons quite different than the well-known ability of this integral membrane protein to give rise to a transient inward current and thus promote cell and/or substrate excitability. The so-called “non-conducting functions” of the Na^+^ channel complex (Kaczmarek, 2006; Brackenbury and Isom, 2011) are gaining increasing attention, for example, in the setting of the metastatic phase of the progression of a number of different solid tumor cancers. During metastasis of solid tumors and during nerve “sprouting,” it is very likely that the expression of Na^+^ channels result in mechanical stability of the cells in which they are expressed, rather than, or in addition to, a conventional electrophysiological function (Chioni et al., 2009; Fraser et al., 2010). Some Na^+^ channel β subunits can covalently link with well-known adhesion molecules. This suggests the possibility that expression of the β subunit, and by implication the entire Na^+^ complex (Abriel and Kass, 2005), may have an important adhesion function. It is interesting that Watanabe et al. (2009) have reported alterations in the β1 and β2 subunits of the Na^+^ channel in tissue taken from atria that had exhibited persistent fibrillation, a condition often associated with enhanced fibrosis and perhaps with altered cell-matrix dynamics.

It is also known that one aspect of an acute inflammatory response involves binding of emigrated neutrophils to the Na^+^ channel complex, and a resulting (very significant) Na^+^ influx, due to a “late” Na^+^ current. This arises from Na^+^ channels opening but then inactivating very slowly (Poon et al., 2001; Ward et al., 2006). Thus, provided that the membrane potential of the myofibroblast is sufficiently negative that even a small fraction of the Na^+^ channels could open (see Figure 2), the pathophysiological chain events involving substantial leak of Na^+^ into the cell through non-inactivating Na^+^ channels followed by Ca^2+^ overload may need to be considered in the setting of cytokine or inflammation-induced apoptosis or necrosis.

Quite recently, the so-called “re-programmed” fibroblast has been advanced as the preparation of choice for heterologous introduction into the compromised myocardium or other end-organs in the context of Regenerative Medicine approaches to Chronic Disease Management (Leri and Kajstura, 2012; Palpant and Murry, 2012; Qian et al., 2012; Song et al., 2012; Srivastava and Ieda, 2012; Miki et al., 2013). The fundamental principles of fibroblast isolation and reprogramming appear now to be well understood. However, in the cases of cardiac and vascular pathophysiology, an essential requirement is that these implanted cells must have a stable, quiescent electrophysiological phenotype. For this reason, the expression of Na^+^ current in myofibroblast that is the focus of this study needs to be understood fully and perhaps avoided or suppressed. Similar considerations are likely to apply to more preliminary attempts to use reprogrammed fibroblasts in the context of cardiac rhythm control (Yankelson et al., 2008; Cho and Marbán, 2010).

### Limitations

A major limitation of the experimental work presented in this paper is that, as judged by both electrophysiological and PCR methodology, expression of Na^+^ channels could be detected only a considerable time period *after seeding* of what were originally human atrial fibroblasts in 2-D tissue culture. This raises the possibility (both with respect to the Chatelier paper and our work) that Na^+^ channel expression could be tissue culture specific. Such an epi-phenomenon would have little if any physiological or pathophysiological significance. Perhaps the strongest argument against this possibility is that, although ion channel expression is known to change in tissue culture, under most circumstances the expression levels are *reduced* (Banyasz et al., 2008). In addition, and as noted in Methods, although the enzymatic isolation of fibroblasts from the human atrial right appendage was somewhat similar in these two studies, in fact, post-isolation treatment of the resulting fibroblast/myofibroblast cell populations differed quite significantly. In our work [consistent with the previous work and ongoing investigations in the Fedak Laboratory (Fedak et al., 2012)], these cells are maintained and held in a 3D-substrate for most of the post-isolation period, i.e., to being released for study by conventional electrophysiological or PCR methods.

The results based on mathematical modeling (Figures 6–9) are preliminary, in part because the experimental data is incomplete. This part of this paper is limited by a lack of information with respect to (i) the extent of the electrotonic coupling between myofibroblasts, and/or (ii) among single or multi-cellular groups of myofibroblasts and the immediately adjacent human atrial myocytes. It would seem implausible that this coupling ratio could be 1:1. Other, more likely possibilities are illustrated in the computations in Figures 7 and 8. It is clear, however, that more extensive and detailed experimental data and more refined computations are needed to further evaluate myocyte/fibroblast electrotonic interactions in defined starting conditions that are directed toward a defined myocyte/fibroblast ratio.

In addition, future work will need to consider the influence of tonic effects of autonomic transmitters and/or paracrine substances. Success based on these improvements may provide the possibility that within the extracellular matrix of the atrium, a “double hit” approach can be developed to modulate Na^+^ channel activity, and thus achieve improved anti-arrhythmic therapy (Maingret et al., 2008).

## Author contributions

Doctors Robert Clark and Darrell Belke were responsible for the experimental work shown in Figures 1–4 and 5, respectively. Doctors Jussi Koivumäki and Mary Maleckar produced the simulation results in Figures 6–9. Doctor Wayne Giles planned and wrote draft #1 of this manuscript. All authors read and provided comments on the original and revised manuscripts.

### Conflict of interest statement

Gilead Sciences provided an unrestricted Grant to Dr. W. Giles. These funds supported part of this study.
